# Unveiling Population Heterogeneity in Health Risks Posed by Environmental Hazards Using Regression-Guided Neural Network

**DOI:** 10.21203/rs.3.rs-8066259/v1

**Published:** 2025-12-22

**Authors:** Jong Woo Nam, Eun Young Choi, Jennifer A. Ailshire, Yao-yi Chiang

**Affiliations:** 1 Neuroscience Graduate Program, University of Southern California; 2 Leonard Davis School of Gerontology, University of Southern California; 3 Department of Computer Science and Engineering, University of Minnesota

## Abstract

As environmental hazards become more frequent, it is critically important to understand their health impacts and identify individuals at disproportionately higher risk. Moderated Multiple Regression (MMR) provides a straightforward approach for investigating population heterogeneity by incorporating interaction terms between hazard exposure and population characteristics into a regression model. However, when vulnerabilities are embedded within complex, high-dimensional covariate spaces, MMR often fails to adequately model complex population heterogeneity. Here, we introduce a hybrid method, Regression-Guided Neural Networks (ReGNN), which integrates the flexibility of artificial neural networks (ANNs) within the structural form of a regression model. Briefly, ReGNN embeds an ANN inside a regression equation to generate a latent representation that nonlinearly combines potential sources of heterogeneity and moderates the effect of an environmental hazard. Because the outer layer maintains a regression structure, the interpretability of standard regression analysis is preserved. Through extensive simulation studies, we demonstrate ReGNN’s effectiveness in modeling complex heterogeneous effects. We further illustrate its utility by applying it to investigate population heterogeneity in the health impacts of air pollution (PM2.5) on cognitive functioning scores. By comparing ReGNN’s results with those from traditional MMR models, we show that ReGNN can uncover patterns of heterogeneity that would otherwise remain hidden.

## Introduction

In 2016, environmental hazards, including air pollution, extreme weather, and harmful chemical exposures, accounted for an estimated 24 percent of global deaths, or approximately 14 million annually. In the United States, ten climate-sensitive events in 2012 resulted in 10.0 billion dollars in health-related costs (2018 dollars)^1,2^. Although these threats are widespread, vulnerable groups, particularly those with limited capacity to anticipate, cope with, or recover from harm, face disproportionate risks^3^. Understanding population heterogeneity in environmental health risks is therefore essential for designing targeted and equitable policies and interventions.

With survey-based population studies becoming increasingly rich and multifaceted, researchers are now better equipped with data to capture a wide range of factors that may differentially influence individual health. Despite these advances, characterizing population heterogeneity remains challenging, as the relevant factors are often complex, and not easily reducible to a single or limited set of measurable variables.

While traditional regression-based approaches offer a straightforward and interpretable way to quantify heterogeneity, they are often inadequate in capturing the multidimensional nature of these relationships. Moderated multiple regression (MMR)^4^, a commonly used method, extends linear regression by adding interaction terms between a focal predictor (i.e. a variable measuring exposure to an environmental hazard) and one or more moderators to assess how effects vary across subgroups. This approach allows researchers to use familiar tools, such as hypothesis testing, to evaluate the statistical significance and magnitude of moderation effects. However, MMR has several limitations: it is prone to structural multicollinearity due to correlations between interaction terms and their components; its statistical power decreases as additional interaction terms are added; and it requires strong prior hypotheses to determine the number and order of interaction terms to include^5^. Consequently, practitioners struggle to model and uncover complex heterogeneity when moderation effect is distributed across multiple variables.

Machine learning approaches, on the other hand, have emerged as promising alternatives to traditional regression-based methods, offering greater flexibility and predictive performance through learning patterns directly from multidimensional data without assuming a fixed functional form. However, this flexibility comes at the cost of interpretability, as most machine learning models do not allow researchers to isolate effect sizes of variables, or their moderating effects, in a transparent way.

For the goal of quantifying effect sizes of variables including their moderation effects, recent developments in explainable AI techniques and in modeling approaches have tried to bridge this transparency gap. Post hoc explainability tools such as SHAP^6^ and LIME^7^ estimate feature importance locally or globally, with the ability to highlight effect modification when the influence of one predictor depends on the level of another.

At the modeling level, interpretable approaches have been developed with the explicit goal of capturing interaction structures that underlie heterogeneity. Generalized additive models with pairwise interactions^8^ extend additive frameworks by including a limited, interpretable set of interactions. Interaction forests^9^, which is a variant of random forests, explicitly detect and exploit interpretable quantitative and qualitative interactions via bivariable splits rather than just axis-aligned univariable splits. Nevertheless, measures derived from these approaches remain difficult to interpret, as their definitions of moderation effects diverge from those in regression. Consequently, they do not yield coefficient estimates with confidence intervals, which are the standard for evaluating the significance of predictors in many fields. As a result, many researchers remain hesitant to fully integrate machine learning models into their analytic workflows when statistical interpretability is prioritized over predictive accuracy.

To addresses the limitations of both traditional regression and machine learning approaches, we propose the Regression Guided Neural Network (ReGNN), a semi-parametric model that effectively captures population heterogeneity by leveraging the flexibility of machine learning while retaining the interpretability of regression. Structurally, ReGNN embeds a neural network within a regression model. Specifically, the neural network first condenses the moderating effect of a set of potential moderators into a single (or a small) dimensional moderator z. Then, the condensed moderator is plugged into a regression model as the sole interaction term, forming a parsimonious MMR model. The coefficients of this outer-layer regression model as well as the parameters of the embedded neural network is optimized using gradient descent to better predict the outcome specified in the MMR.

Embedding the neural network within an MMR constrains it to learn additional nonlinear or higher-order moderation effects that are not already explained by predictors’ main effects. As a result, the interaction effects captured by ReGNN remain formulaically consistent with those used in traditional regression analysis. Moreover, standard statistical tools, such as coefficient estimates and confidence intervals, remain applicable within this framework. This enables the condensed moderator learned by ReGNN to be interpreted within a familiar statistical paradigm, allowing researchers to quantify contributions to effect heterogeneity using conventional inferential metrics.

At a high level, ReGNN scores each individual’s vulnerability to the studied environmental hazard, inferred from input variables. In other words, it stratifies the population by how exposure effects are amplified or reduced by individual characteristics. Unlike MMR, which requires moderators to be explicitly specified in advance, ReGNN learns a composite moderator from potentially high-dimensional covariates, enabling detection of complex and diffuse patterns of effect heterogeneity that traditional regression cannot capture. Taken together, ReGNN offers a framework that combines the flexibility of machine learning with the interpretability of regression, well-suited for studies where quantifying and understanding population heterogeneity is critical.

## Results

### Regression-Guided Neural Network Framework

Here, we introduce ReGNN and the general analytic workflow using to model and uncover population heterogeneity. Moderated multiple regression is a simple extension of linear regression that allows one to capture systematic differences in how a focal predictor affects an outcome through addition of interaction terms:

(1) $$o=c_{0}+\sum_{k=1}^{n-1}c_{k}x_{k}+c_{n}x_{f}+\sum_{k=1}^{j}c_{k}^{int}x_{k}x_{f}$$

where ${\text{x}}_{\text{f}}$ is the focal predictor, ${\text{c}}_{\text{k}}$ are the coefficients for the independent variables ${\text{x}}_{\text{k}},{\text{c}}_{\text{n}}$ is the regression coefficient for ${\text{x}}_{\text{f}}$, and ${\text{c}}_{\text{k}}^{\text{int}}$ are regression coefficients for interaction terms ${\text{x}}_{\text{k}}{\text{x}}_{\text{f}}$. In practice, however, throwing in all remaining predictors ${\text{x}}_{\text{k}}$ in the interaction terms causes multicollinearity and reduction of the model’s statistical power, making it difficult to find any meaningfully significant moderation effect^5^. Additionally, the true moderation effect may involve a higher-order interaction with other predictors ${\text{x}}_{\text{k}}$, which the equation (1) does not capture.

To overcome these limitations, ReGNN leverages neural networks to create a parsimonious regression model with just a single (or a few) interaction term. Suppose we take the equation (1) and modify it by replacing all interaction terms with a single term that multiplies the focal predictor ${\text{x}}_{\text{f}}$ with an output of a neural network:

(2) $$o=c_{0}+\sum_{k=1}^{n-1}c_{k}x_{k}+c_{n}x_{f}+c^{int}f\left(X_{p}\right)x_{f}$$

where f represents the function approximated by the neural network, ${\text{c}}^{\text{int}}$ is a regression coefficient for the replaced interaction term, and ${\text{X}}_{\text{p}}$ is a vector representation of inputs to the neural network (${\text{x}}_{1}\ldots {\text{x}}_{\text{j}}$ in equation (1)), which are potential sources of heterogeneity. We employ gradient descent to minimize the mean squared error (MSE) objective for the outcome o, jointly optimizing the regression coefficients and the neural network parameters (Figure 1, step 1).

After arriving at a suitable f, the neural network is fixed $\left(\hat{\text{f}}\right)$ and used to generate the condensed moderator $\text{z}=\overset{ˆ}{\text{f}}\left({\text{X}}_{\text{p}}\right)$. We then fit a “twin” regression model, which follows the same form as equation (2), except that $\text{f}\left({\text{X}}_{\text{p}}\right)$ is replaced by z (Figure 1, step 2). This model allows us to compute regression coefficients, confidence intervals, and statistical significance for all terms, including the interaction term, $\text{z}{\text{x}}_{\text{f}}$.

Interestingly, we find that this structural constraint guides the neural network to learn a meaningful reduced-dimensional representation that significantly moderates the focal predictor’s effect. While stochastic gradient descent may yield regression coefficients that differ from the closed-form estimates of traditional regression due to batch sampling noise, we observe that the overall magnitude of the coefficients (L2-norm) estimated in Step 1 stabilizes as training progresses. Correspondingly, the outcomes predicted in Steps 1 and 2 become highly correlated once the neural network converges. This indicates that the MSE objective, combined with the structural constraint imposed by the outer regression layer, effectively guides ReGNN to learn both a composite moderator and a set of regression coefficients that predict the outcome with reasonable accuracy. We therefore term this approach the Regression-Guided Neural Network, as its neural network component is trained via a regression loss that serves as a proxy supervisory signal, unlike conventional supervised algorithms, where minimizing MSE is both the means and the objective.

ReGNN offers several improvements to MMR. First, because the neural network figures out all higher-order interactions with the focal predictor from the data rather than fitting the data to a pre-specified equation, ReGNN frees practitioners from having to guess which, how many, and which order of interaction terms to include in their model. Second, embedding the neural network within ReGNN’s regression outer layer aligns its definitions of moderation effects to that of MMR as measured by how much the marginal effect of ${\text{x}}_{\text{f}}$ changes according to different levels of z:

(3) $$\frac{\partial }{\partial x_{f}}E\left[o|x_{f},z=z^{\prime },x_{1},\ldots ,x_{n-1}\right]=c_{n}+c^{int}z^{\prime }$$

where the left-hand side of equation (3) denotes the partial derivative of the conditional expectation of the outcome with respect to ${\text{x}}_{\text{f}}$, evaluated at an arbitrary level ${\text{z}}^{\prime }$. Unlike non-parametric machine learning models, moderating effects of ${\text{x}}_{1},\ldots ,{\text{x}}_{\text{j}}$ (potential sources of heterogeneity) on ${\text{x}}_{\text{f}}$ are constrained to happen through ReGNN’s interaction term $\text{f}\left({\text{X}}_{\text{p}}\right){\text{x}}_{\text{f}}$. As a result, the moderating effect size can be quantified directly using ${\text{c}}^{\text{int}}$, rather than relying on derived measures such as partial dependence.

Once a significant moderation effect of z is established, the final step is to interpret what the neural network has learned (Figure 1, step 3). To achieve this, we conduct post-training analyses that use tools, such as feature importance measures^6^, to identify which input variables contribute most to the construction of z. In addition, we apply unsupervised clustering methods, such as Gaussian Mixture Model (GMM), to group individuals with similar vulnerability profiles. These complementary approaches allow researchers to visualize and understand the sources of effect heterogeneity captured by ReGNN, providing critical insight for scientific interpretation.

### Simulated study

The primary goal of ReGNN is to consolidate the moderating influence of potentially high-dimensional inputs into a single summary variable that modifies the effect of a focal predictor, thereby aiding the discovery of hidden population heterogeneity. ReGNN is therefore best understood as an explanatory model, akin to regression, rather than a predictive model, where out-of-sample predictive accuracy is the primary objective^10^. Consequently, the significance of the ReGNN-derived moderation effect is assessed through the p-value of the interaction coefficient ${\text{c}}^{\text{int}}$. However, because z is not measured but learned, gradient descent optimizes z to maximize its explanatory power for o, often leading to a statistically significant ${\text{c}}^{\text{int}}$ by construction. This raises important questions for practitioners regarding whether, and to what extent, the learned summary variable captures true underlying moderation effects rather than spurious patterns from noise.

To address this concern, we evaluate ReGNN’s ability to recover true moderation effects using a simulated dataset with a predefined, complex moderation structure. In parallel, we (1) develop a sensitivity metric to evaluate ReGNN’s capacity to recover moderation structures in applied settings where the ground truth is unknown, and (2) examine how the recovery accuracy influences post-training analyses, thereby facilitating greater confidence in ReGNN-based analysis framework.

As with other nonparametric estimators, the performance of ReGNN in accurately recovering the ground truth depends on several factors, including the dimensionality of the input variables, the smoothness of the ground truth function, parameters of the estimator, and the magnitude of measurement noise^11^. While input dimensionality can be reduced through careful selection of input variables and model parameters can be adjusted to accommodate complex functions, measurement noise in real world datasets is often unavoidable. Therefore, we focus our experiment on examining how the recovery accuracy of ReGNN varies under different signal-to-noise ratios (SNRs) computed as the ratio of the variance of the ground truth moderator M to the variance of the measurement noise Var(M)σnoise2.

To this end, we generate a dataset of 8,000 samples with 31 variables. All variables are initially continuous, drawn from a multivariate normal distribution. We then transform them to create 7 binary, 5 categorical, and 3 ordinal variables, while retaining 16 as continuous. A subset of these variables is then randomly selected to define the ground truth moderator M, which interacts with the focal predictor ${\text{x}}_{\text{f}}$ through a complex moderation structure, defined by a 3^rd^ degree polynomial. The outcome variable o is generated according to the following model:

(3) $$o=\sum_{k=1}^{30}c_{k}x_{k}+c_{f}x_{f}+Mx_{f}+\epsilon$$

where ${\text{c}}_{\text{k}}$ and ${\text{c}}_{\text{f}}$ are linear coefficients for predictors ${\text{x}}_{\text{k}}$ and the focal predictor ${\text{x}}_{\text{f}}$ respectively, and ϵ is measurement noise $\left(\sim \text{N}\left(0,\sigma^{2}\right)\right)$. We generate multiple outcome variables using varied noise variance $\left(\sigma^{2}\right)$, which we utilize to quantify the effect of SNR on ReGNN’s recovery accuracy. Further detail about how we construct the simulated dataset can be found in the methods section.

We then train ReGNN separately on each outcome with varying levels of measurement noise. For comparability, hyperparameters are held constant across all experiments. ReGNN’s recovery accuracy is quantified using the Pearson correlation between the neural network output z and the ground-truth moderator M.

In addition, we use 80% of the dataset for training and evaluate the statistical significance of the ReGNN-derived summary variable z on a small sensitivity test set comprising 2% of the data. Although splitting data is uncommon in explanatory modeling, we introduce this sensitivity set to use the p-value of its interaction term as a sensitivity probe. Because the p-value depends on both effect size and sample size, evaluating on a very small subset provides a stringent test of the stability of the learned moderator. If the interaction between z and the focal predictor remains statistically significant despite the reduced sample size and hence wider confidence intervals on unseen data, it suggests that ReGNN has captured a strong and stable moderation structure. Conversely, if significance diminishes on this sensitivity test, it indicates that the detected moderation effect on the train set is weak.

**Figure 2.**
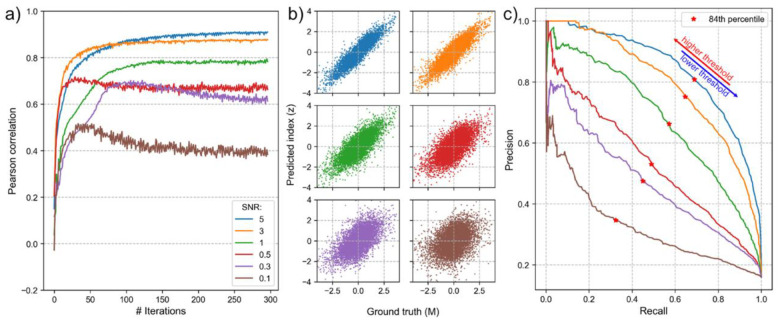
**(a)** Trajectories of correlation values between the predicted vulnerability score z produced by ReGNN and the ground truth moderator M over the course of training, shown across different signal-to-noise ratios (SNRs). SNR is defined as the variance of M divided by the variance of the noise term ϵ. As SNR decreases, correlation between z and M achieved at the end of training diminishes. **(b)** Scatter plot of M vs z at the end of training. **(c)** Precision-recall curves for identifying the vulnerable population based on varying thresholds z, relative to the true subgroup defined as $\text{M}>84^{\text{th}}$ quantile, across SNRs.

Figure 2 and Table 1 summarize how different SNR levels affect ReGNN’s recovery accuracy. Figure 2a and 2b depict the trajectories of Pearson correlations between the neural network’s output z and the ground truth moderator M across SNRs ranging from 0.1 to 5, along with scatter plots of M and z at the end of training. Table 1 reports correlations between M and z(ρ) achieved at the end of training, as well as root-mean-squared error (RMSE) and p-values of the interaction term $\text{z}{\text{x}}_{\text{f}}$ from regression models estimated on both training and sensitivity sets. ReGNN performs well on recovering the ground-truth moderator in low measurement noise regime, achieving correlation ~0.8 or above (SNR ≥ 1). The correlation decreases as the signal conveyed by the moderator weakens relative to noise, as expected (Figure 2a).

How can we quantify ReGNN’s recovery accuracy in practice, when the ground-truth is unknown? RMSE serves as a useful proxy for model performance (Table 1), increasing as the correlation declines in both training and sensitivity sets. However, because there is no absolute benchmark for RMSE, it is most appropriately used for relative comparisons across ReGNN models applied to the same outcome and focal predictor under varying hyperparameter settings, rather than as a measure of absolute accuracy. On the other hand, the p-value of the interaction term $\text{z}{\text{x}}_{\text{f}}$ on the sensitivity set offers a clearer interpretive utility, as it has a well-established threshold for statistical significance. As shown in Table 1, while the p-value for the training set remains near zero even as the correlation between z and M declines, the sensitivity set’s p-value is much more sensitive to decreases in model performance. In particular, it rises above 0.05 – a commonly used significance threshold – when the correlation between z and M falls below 0.6.

Analytically, this behavior arises because the t-statistic underlying the calculation of p-value depends on the sample size, as well as implicitly incorporating the signal-to-noise ratio (SNR):

(4) $$t=\frac{c^{\text{int}}}{\text{SE}\left(c^{\text{int}}\right)}\approx c^{\text{int}}\frac{s^{\text{int}}}{\sigma_{\text{residual}}}\sqrt{n}$$

where $\text{SE}\left({\text{c}}^{\text{int}}\right)$ is the standard error, governed by the ratio of the independent variance of the interaction term $\left({\text{s}}^{\text{int}}\right)$ to the residual variance $\left(\sigma_{\text{residual}}\right)$ and the square root of the sample size (n). Here, ${\text{s}}^{\text{int}}/\sigma_{\text{residual}}$ can be viewed as an empirical estimate of 1/SNR. Consequently, by examining how the p-value changes on subsets with different sample sizes of unseen data, we can indirectly assess the underlying SNR, which govern the recovery accuracy of ReGNN. We note that although we use a sensitivity set size of 2% in this simulation, practitioners can adjust its size, or use multiple sets of varying size, as a practical guideline for gauging ReGNN’s recovery accuracy, depending on the characteristics of their data.

How does ReGNN’s recovery accuracy in modeling the ground-truth moderator influence downstream post-training analyses? Although higher accuracy in recovering M is desirable, the primary scientific aim of ReGNN is to identify vulnerable subgroups and characterize their features (step 3, Figure 1). To examine this, we evaluate how two post-training procedures, SHAP-based feature importance and GMM clustering, are affected by ReGNN’s recovery accuracy. SHAP values are used to globally rank the importance of input features, while GMM is applied to the vulnerable subpopulation, identified by thresholding on the levels of z, to cluster individuals into subgroups with similar vulnerability profiles.

**Figure 3.**
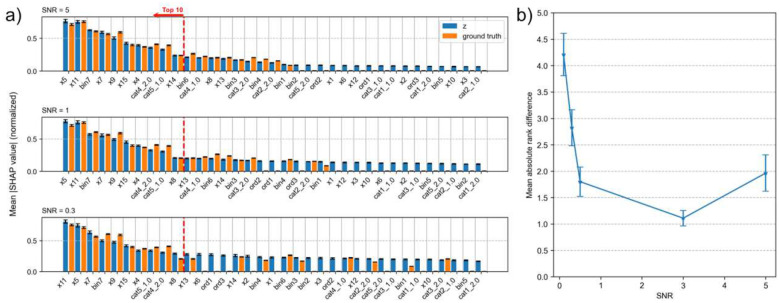
Comparison of feature importances (mean absolute SHAP values) for the ground-truth moderator and ReGNN-derived index across different SNR levels. **a)** Bar plots of mean absolute SHAP values for each feature, in descending order of importance predicting z. **b)** Mean absolute rank difference between top 10 features for ReGNN-derived index (z) and ground-truth moderator (M), by SNR. The rank discrepancies do not differ significantly for SNRs > 0.3.

To assess the stability of SHAP-based feature importance rankings, we apply repeated cross-validation and compute confidence intervals around the mean absolute SHAP values. We further elaborate how cross-validation is done in methods section. A similar procedure is used to estimate SHAP-based feature importances for the ground-truth moderator M. Figure 3a presents each feature’s mean absolute SHAP value, ordered by importance in predicting z (blue), across varying SNR levels. Error bars represent 95% confidence intervals across repeated resampling. For comparison, mean absolute SHAP values for predicting the ground-truth moderator M are shown in orange. To facilitate visual comparison, mean absolute SHAP values for z and M are normalized by dividing each by the maximum SHAP value across all samples from cross-validation. We repeat this procedure for all SNRs. We plot these normalized mean absolute SHAP values for SNR=5, 1, 0.3 in Figure 3a.

Figure 3a shows that across different levels of SNR, the SHAP-based feature importance ranks of z (blue) do not differ significantly to that of the ground-truth (orange), especially for features that rank higher. To quantify this stability, we identify the top 10 features predicting z and compute their rank discrepancies relative to their importance ranks in predicting M. Figure 3b reports the mean absolute rank difference across these top 10 features, computed for each sample and averaged over all samples from cross-validation. We observe that the mean absolute rank difference remains stable for SNR values between 5 and 0.5 but increases when SNR drops below 0.5. In other words, although ReGNN’s recovery accuracy, as measured by the correlation between z and M, declines substantially from SNR = 5 (ρ=0.911) to SNR = 0.5 (ρ=0.613), the SHAP-based feature rankings remain largely unaffected.

On the other hand, the mean absolute rank difference increases when SNR < 0.5. Examining the underlying SHAP values reveals that, under high-noise conditions (e.g., SNR = 0.3; Figure 3a), the SHAP values of less relevant features become inflated. This inflation causes lower-ranked features to shift in order, resulting in greater instability in feature rankings. These results suggest that, in high-noise settings, features with substantially larger SHAP values relative to others can still be considered reliable, whereas smaller differences in SHAP values should be interpreted with caution.

Another post-training analysis conducted as part of the ReGNN framework involves clustering the vulnerable population identified by ReGNN into subgroups with similar characteristics. Briefly, we fit a Gaussian Mixture Model (GMM) to the ReGNN-identified vulnerable population, using both the original input features to the neural network and the ReGNN-derived index z as inputs. To evaluate how ReGNN’s recovery accuracy affects the identification of the vulnerable population, which is the first step of the clustering analysis, we plot a precision-recall curve (Figure 2c) across varying threshold levels applied to z. The ground-truth vulnerable population is defined as individuals exceeding the 84th percentile (approximately one standard deviation above the mean) of M. The corresponding recall value obtained using the same percentile threshold for z is reported in Table 1.

Table 1 shows that recall declines as measurement noise increases, meaning that the ReGNN-identified vulnerable population captures a smaller share of the ground-truth vulnerable group. Since the goal is to identify and characterize vulnerable individuals, lower thresholds could be adopted to increase recall (Figure 2c). However, achieving good clustering performance requires balancing recall with precision. If the identified vulnerable group contains more false positives than true positives (i.e., low precision), the resulting clusters may shift in distribution and deviate from the ground truth.

To examine how precision-recall trade-offs affect clustering performance, we evaluate the impact of applying different threshold levels (70th ~ 95th percentiles in z) to z on the GMM results. Procedurally, we first fit a GMM to the ground-truth vulnerable population (M>0.84 quantile), identifying four clusters. These clusters serve as the reference. We then fit a second GMM to the ReGNN-identified vulnerable population, defined by the chosen threshold, fixing the number of components at four to enable one-to-one alignment with the reference clusters. Cluster-to-cluster correspondence is established by minimizing the total Mahalanobis distance between matched pairs of clusters. Finally, we assess the sensitivity of the clustering results by examining how these Mahalanobis distances vary across threshold levels and SNR conditions.

**Figure 4.**
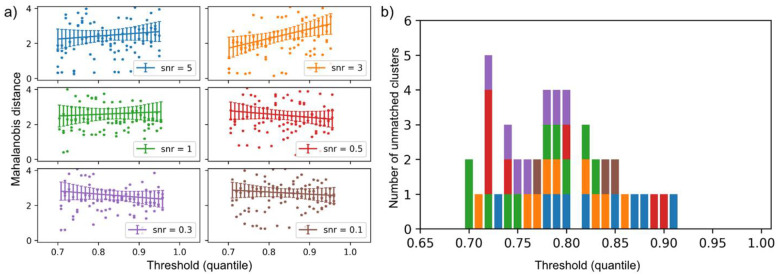
Mahalanobis distances to reference clusters for vulnerability scores fit on datasets with varying SNRs. Clusters are matched to reference clusters by minimizing overall distance. Clusters with distances greater than 10 are excluded from panel (a) and counted separately in panel (b). Lines in panel (a) represent linear fits of threshold versus distance, with error bars showing 95% confidence intervals.

Figure 4a shows the Mahalanobis distances between the reference clusters and their corresponding clusters from the GMM applied to the ReGNN-identified vulnerable population across different threshold levels. Clusters with distances greater than 10 are excluded from the plot and tallied separately in Figure 4b. Distances below 10 are shown as scatter points, and a linear model is fitted to illustrate the overall relationship between Mahalanobis distance and threshold level. Overall, between-cluster distances are noisy and exhibit no clear patterns; however, the fitted linear trends reveal some discernible tendencies. When SNR ≥ 1, distances decrease slightly at lower thresholds, likely because a broader portion of the ground-truth vulnerable group is captured without substantial loss in precision. In contrast, when SNR < 1, distances increase as thresholds are lowered, indicating that maintaining higher precision is more advantageous under noisier conditions. Figure 4b further shows that the number of unmatched clusters does not follow a clear pattern across thresholds or SNR levels. In practice, these findings underscore the importance of testing multiple threshold values to obtain stable insights and effectively filter out outlier clusters.

In summary, our simulated study shows that ReGNN effectively learns a representation that interacts with the focal predictor, enabling the recovery of underlying moderation structures. While ReGNN, like other estimators, is affected by measurement noise, we have shown that the degree of this influence is both detectable through the proposed sensitivity test and does not substantially degrade downstream analyses, where key scientific insights are derived.

### Cognition vs air pollution: ReGNN uncovers moderation effect not discovered by traditional regression

We demonstrate ReGNN’s utility in real-world contexts by applying it to assess how air pollution differentially affects cognitive functioning in older adults.

We compile four primary datasets for our analysis. First, we draw on individual-level data from the 2016 Health and Retirement Study (HRS), a nationally representative survey of U.S. adults aged 50 and older^12^. These data provide extensive sociodemographic and health information, including our outcome measure (cognitive functioning score). Second, we incorporate 2016 daily fine particulate matter (PM2.5) concentration estimates from the U.S. Environmental Protection Agency’s Fused Air Quality Surface Using Downscaling (FAQSD) files, which employ a data fusion, or “downscaler,” model to generate local PM2.5 concentrations across the contiguous U.S^13^. To capture short-term exposure, we average PM2.5 concentrations over the 30 days preceding each respondent’s interview date. Third, we use tract-level socioeconomic indicators from the U.S. Census Bureau’s 2014–2018 American Community Survey (ACS) 5-year estimates^14^. Both the PM2.5 and ACS datasets are available through the HRS Contextual Data Resource and are linked to the 2016 HRS sample using respondents’ geocoded census tract and interview dates. Finally, we obtain tract-level land cover measures from the U.S. Geological Survey’s National Land Cover Database, accessed via the National Neighborhood Data Archive^15^, and link these to the 2016 HRS records. Further detail about each dataset is provided in the Methods section.

**Figure 5.**
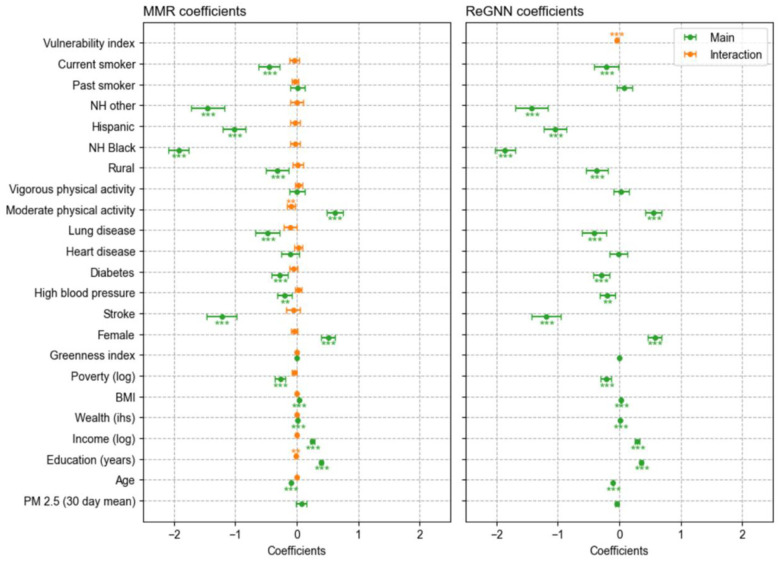
Regression coefficients comparing MMR models with (left) all predictors included as moderators (adjusted r2=0.2953) and (right) only output of the trained neural network, which we name vulnerability index, included as moderator (adjusted r2=0.2955). The position along the x-axis tells each coefficient’s value, along with its confidence interval, which is indicated by the error bar. Significance levels are indicated with asterisks (***:p<0.001;**:p<0.01;*:p<0.05)

Compared with MMR model, ReGNN demonstrates greater capacity to identify population heterogeneity that is otherwise difficult to detect. Figure 5 illustrates this by comparing the regression coefficients of MMR and ReGNN’s twin regression model. The MMR model includes all predictors as moderators, and whereas the twin-regression model only has ReGNN-derived index z as the sole moderator of PM2.5. In All predictors are included as linear terms in both. Whereas most interaction terms in the MMR model are non-significant with only a weak association shown for education and moderate physical activity, ReGNN-derived vulnerability index moderates the effect of PM2.5 with high statistical significance (p < 0.001). Additionally, because the vulnerability index combines and summarizes the moderation effect across predictors nonlinearly, multicollinearity is reduced: Variance inflation factors (VIFs) are higher for the traditional MMR model (maximum VIF = 12.01 for PM2.5, mean centered) than for the twin-regression model incorporating z (maximum VIF = 3.23 for the interaction term).

**Figure 6.**
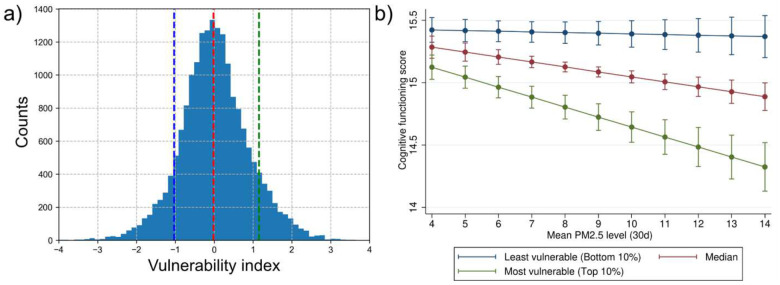
(a) Histogram of the ReGNN-derived vulnerability index z. (b) Predicted cognitive functioning scores from an MMR model using the ReGNN-derived vulnerability index z as the sole moderator of PM2.5. All other predictors are held at their means. The fitted MMR is used to estimate mean cognitive scores and 95% confidence intervals across varying levels of PM2.5. Results are shown separately for groups with low (bottom 10th percentile), median, and high (top 10th percentile) values of the vulnerability index, illustrating its moderating effect on the PM2.5–cognition association.

Although the regression coefficient of this index may look small (Figure 5, top row, orange), its moderating effect is quite significant, as displayed in Figure 6b. Here, we plot the means and confidence intervals of the predicted cognitive functioning scores using the fitted twin regression model, for differing levels of PM2.5. Other independent variables are held at their means in getting the predicted cognitive scores. We observe that the deteriorating health effect of air pollution on cognition is significantly reduced for those with a lower vulnerability index (blue, bottom 10th percentile) while it is inflated for those who score higher on the vulnerability index (green, top 10th percentile). In other words, exposure to higher levels of air pollution leads to a measurable decline in cognitive function among the vulnerable group, whereas the effect is minimal among the more resilient group. Because the error bars for each predicted point do not overlap across the three groups, we conclude that the vulnerability index significantly moderates the effect of PM2.5 on cognition.

**Figure 7.**
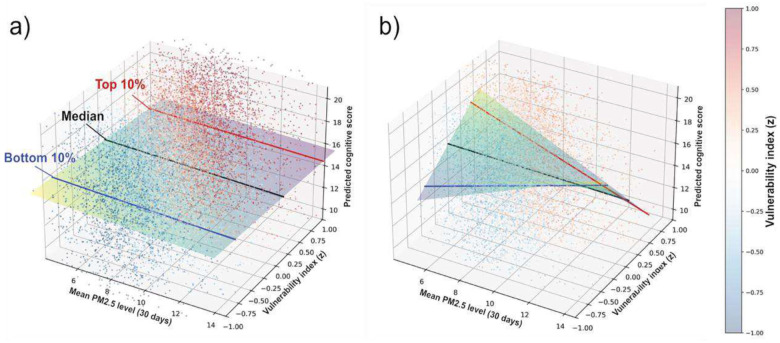
Comparison of fitted interaction surfaces estimated at the beginning of training (a) and after convergence (b). The plots show the fitted MMR surface ($\text{o}={\text{c}}_{\text{int}}\cdot {\text{x}}_{\text{f}}\cdot \text{z}$, linear terms controlled), along with three lines representing the 10th, 50th, and 90th percentiles of ReGNN-derived index z. (a) Prior to training, z is randomly initialized, resulting in similar slopes across the three lines and no meaningful moderation effect. (b) After training, the ReGNN-derived index z effectively stratifies the population, with the three lines exhibiting markedly different slopes, indicating a statistically significant moderation effect. Because individuals’ vulnerability to the focal exposure ${\text{x}}_{\text{f}}$ varies along this dimension, we refer to z as “vulnerability index”. Data points are colored according to their vulnerability indices (red to blue).

The ReGNN-derived index z can be viewed as a dimension that stratifies the population according to each individual’s predicted vulnerability, based on the inputs to its neural network. Geometrically, fitting a regression model with an interaction term can be interpreted as fitting a surface $\text{o}={\text{c}}^{\text{int}}\cdot {\text{x}}_{\text{f}}\cdot \text{Z}$ to the data. Figure 7 visualizes this fitted surface by plotting the outcome (o), focal predictor ${(x}_{f})$, and moderator (z) along orthogonal axes, both at the start of training (Figure 7a) and after training has converged (Figure 7b). The three lines (blue, black, and green) correspond to the 10th, 50th, and 90th percentiles of z and are equivalent to those shown in Figure 6b.

At the beginning of training, populations separated along the vulnerability dimension do not exhibit diverging trends in the $\text{o}-{\text{x}}_{\text{f}}$ plane, resulting in fitted lines that are nearly identical across all levels of zand a flat fitted surface (Figure 7a). As training progresses, ReGNN learns to produce a representation that meaningfully stratifies the population, yielding a learned z that separates groups with diverging outcome trends when projected onto the $\text{o}-{\text{x}}_{\text{f}}$ plane. Put differently, ReGNN condenses moderation effects from across all input features into a single dimension, even when the individual moderation effects of those features are weak or insignificant when considered in isolation (left, Figure 5, orange points).

Because the slopes of the fitted $\text{o}-{\text{x}}_{\text{f}}$ relationships differ markedly across levels of z, this dimension can be interpreted as an outcome- and hazard-specific “vulnerability index”. As shown in Figure 6b, individuals with higher vulnerability indices experience more adverse effects from exposure to the environmental hazard under investigation, with respect to the studied outcome.

With regression coefficients showing a significant moderation effect of the vulnerability index, it remains now to understand how ReGNN produces them. To this end, we employ explainable AI tools, mainly SHAP values and GMM-based clustering, to parse which predictors contributed to the network’s assignment of values for this index.

Figure 8a presents the feature importance rankings of potential moderators used as inputs to the trained ReGNN. Education emerges as the most influential factor, consistent with its prominent interaction coefficient in the MMR model (left, Figure 5). However, the interaction coefficient for education is negative, suggesting that individuals with more education appear more vulnerable to PM2.5 exposure, when modelled with MMR. In contrast, ReGNN indicates the opposite effect: partial dependence plot in Figure 8b shows that the vulnerability index declines as education increases. This discrepancy highlights how considering a single variable in isolation, as in MMR, can lead to misleading conclusions, as it aggregates the moderation effect over the whole population.

Table 2 summarizes the top eight features of clusters identified by GMM fit to the vulnerable subgroup, defined using the 0.9 quantile threshold. Overall, lower education and wealth, coupled with higher poverty, emerge as key drivers of vulnerability. Smoking status also contributes. Both current and former smokers are more likely to be classified as vulnerable. Finally, a higher proportion of females is observed among the vulnerable subgroups. We conduct a review of relevant literature to assess how aligned the high-ranked features are with the past findings. We find that the directions of the influences for many features are aligned: people who received 8 years or less education are found to have higher probability of cognitive impairment with increased PM2.5 exposure^16^; individuals who smoked daily are at higher risk of dementia when exposed to higher concentration of PM2.5^17^; women are at a higher risk for decreased cognitive function associated with increased exposure to PM10 and PM2.5–10^18^;

## Discussion

The Regression-Guided Neural Network (ReGNN) provides a powerful new framework that integrates the predictive flexibility of neural networks with the interpretability and inferential rigor of regression. In this study, we introduce its simplest form, which leverages a neural network to condense complex moderation patterns into a single, interpretable dimension, which we call the vulnerability index. Through both simulation and application to real-world data, we demonstrate that this ReGNN-derived index accurately models the underlying moderation structure and reveals meaningful population heterogeneity that would remain hidden using traditional methods alone.

This capability is particularly timely for environmental health research. As climate change and urbanization intensify the frequency and severity of environmental hazards, identifying vulnerable subpopulations has become increasingly urgent for informing equitable policy and intervention strategies. Beyond environmental health, ReGNN’s flexibility and interpretability make it broadly applicable to any domain where understanding population heterogeneity is essential while preserving the familiar inferential framework of regression.

Beyond its current formulation, the underlying concept of embedding a machine learning model within a regression framework offers substantial structural flexibility. This approach is not limited to linear regression and can, in principle, be extended to a wide range of statistical models. For example, it could be adapted to generalized linear models to accommodate nonlinear outcomes, to multilevel or hierarchical models to capture nested data structures, or to longitudinal models to examine time-varying moderation effects. In addition, the framework can be used to learn optimal transformations of covariates, such as identifying thresholds or nonlinear functions of temperature to best model its relationship with an outcome, when their functional forms are unknown.

In summary, ReGNN unites the interpretability of regression with the flexibility of neural networks, offering a powerful new framework for investigating population heterogeneity. By uncovering complex moderation patterns while retaining familiar inferential tools, ReGNN addresses a critical methodological gap in contemporary data analysis. As the demand for scalable, interpretable, and rigorous analytic approaches continues to grow, ReGNN provides a versatile foundation for both methodological innovation and substantive applications across diverse domains.

## Methods

### Simulated Study

#### a) Constructing Simulated Dataset

We randomly generate a dataset with 8,000 samples of 31 variables drawn from a multivariate normal distribution N(0,Σ), where Σ is a 31 by 31 matrix. Its diagonal elements $\sigma_{\text{kk}}$ are set to one, and 60 percent of the off-diagonal elements $\sigma_{\text{kl}}(\text{k}\neq \text{l})$ are drawn from Unif(−0.3,0.3), while the rest of the off-diagonal elements are set to zero. This structure is designed to reflect correlations among variables as seen in real-world datasets. After constructing the matrix, we adjust its eigenvalues to ensure that Σ is positive semidefinite.

All 31 variables are initially sampled as continuous. Of these, 16 are retained as continuous, while 7, 5, and 3 are transformed into binary, categorical, and ordinal variables, respectively, through threshold-based post-processing. Binary and categorical variables are then one-hot encoded, with one category designated as the reference group. One of the continuous variables is picked to serve as the focal predictor ${\text{x}}_{\text{f}}$. Unlike typical causal inference settings that assume random assignment of the focal predictor (also known as the “treatment” variable), we intentionally draw ${\text{x}}_{\text{f}}$ from the same multivariate normal distribution to make it correlated with other variables, as is common in most survey-based studies. We note that ReGNN captures population heterogeneity effectively regardless of whether the focal predictor is correlated with other variables.

We then select subsets of the 30 variables to construct a complex moderation pattern. Specifically, we randomly sample, with replacement, five pairs and five triplets and use them to define the ground truth moderation term:

$$M=\sum c_{\text{pair}}x_{i}x_{j}+\sum c_{\text{tri}}x_{i}x_{j}x_{k}$$

The weights for each term $\left({\text{c}}_{\text{pair}},{\text{c}}_{\text{tri}}\right)$ are also randomly drawn from Unif(0.05,0.3), with their signs randomly selected.

Finally, we generate the outcome variable o using the following model:

$$o=\sum_{k=0}^{30}c_{k}x_{k}+c_{f}x_{f}+Mx_{f}+\epsilon$$

where ${\text{c}}_{\text{k}}$ and ${\text{c}}_{\text{f}}$ are linear coefficients for predictors ${\text{x}}_{\text{k}}$ and the focal predictor ${\text{x}}_{\text{f}}$ respectively, and ϵ is random sampling noise $\left(\sim \text{N}\left(0,\sigma^{2}\right)\right)$. All coefficients are drawn randomly $\left({\text{c}}_{\text{k}},{\text{c}}_{\text{f}}\sim \text{N}(0,0.3)\right)$. We scale the variance of the sampling noise according to the desired signal-to-noise ratios (SNRs) relative to the variance of the ground-truth moderator M.

#### b) ReGNN training parameters

We implement a ReGNN with a multi-layer perceptron (MLP) of size (35, 60, 32, 1) embedded within a regression model. We train the network for 100 epochs with a batch size of 50. Note that we observe experimentally that smaller batch sizes yield higher recovery accuracy. A dropout rate of 20% is applied during training. We use distinct learning rates for the regression coefficients (lr = 0.01) and for the MLP parameters (lr = 0.003); the faster rate for the regression coefficients enables quicker convergence of the outer layer, allowing the embedded neural network to learn under constraint. Of the 8,000 total samples, 80% serve as the training set and 2% as the sensitivity set. All model training implementation is done using Pytorch^19^, while model fitting and evaluations of regression models are done using Stata^20^.

#### c) SHAP-based feature importance

We compute Shapley Additive Explanations (SHAP) using the Python *shap* library^6^. To evaluate the stability of SHAP-based importance measures, we split the dataset into five folds and train ReGNN five times, reserving each fold once as the test set. For each trained model, we draw 500 background samples from the training set and 100 evaluation samples from the test set to compute SHAP values. This computation is repeated 10 times per model. We then calculate mean absolute SHAP values for the 100 evaluation samples, yielding 50 estimates per feature (5 models × 10 repetitions). From these, we derive 95% confidence intervals, shown as error bars in Figure 3a. The same procedure is used to compute SHAP-based feature importances for the ground-truth moderator M.

#### d) Gaussian Mixture Model

We fit Gaussian Mixture Models (GMMs) using the Python *scikit-learn* library^21^. To establish one-to-one correspondence between the reference clusters (from the GMM fit to the ground-truth vulnerable subgroup) and the clusters identified by ReGNN, we compute Mahalanobis distances for all cluster pairs. Distances are calculated using a covariance matrix obtained by averaging the covariance matrices of the two clusters being compared. Cluster pairs are then matched by minimizing the total Mahalanobis distance. Finally, we fit linear models predicting Mahalanobis distance as a function of threshold using the Python *statsmodels* library^22^.

### Cognition vs PM2.5 study

#### a) Dataset

##### Health outcome: cognitive functioning score

HRS respondents’ cognitive functioning is assessed using the modified Telephone Interview for Cognitive Status (TICSm)^23^. The TICSm evaluates three cognitive domains: memory (test of 10 words immediate and delayed recall, range: 0–20 points), working memory (test of serial 7s subtraction, 0–5 points), and speed of mental processing (test of backward counting, 0–2 points). The composite summary score ranges from 0 to 27.

##### Focal predictor: Air pollution (PM2.5)

For each HRS respondent, we calculate the mean concentration of residential PM2.5 (μgm3) at the census tract level over the past 30 days preceding their interview dates.

##### Other input variables

Other predicting and moderating variables are selected a priori, based on their potential roles as effect modifiers in the association between air pollution and cognitive function^24^ as well as their roles as main risk (or protective) factors for the cognitive function.

Individual-level predictors include age, gender (men and women), race/ethnicity (non-Hispanic White, non-Hispanic Black, Hispanic, and non-Hispanic Other), education years, household income (log-transformed), household non-housing financial wealth (transformed using an inverse hyperbolic sine), BMI index, five self-reported chronic conditions diagnosed by a physician (i.e., stroke, heart disease, lung disease, diabetes, hypertension), depressive mood, light, moderate, and vigorous physical activity levels (never, <1 per month, 1–3 times per month, 1–2 times per week, and 3+ times per week).

Area-level predictors include urban/rural residence, and green space index. Green space within each census tract is quantified by calculating the percentage of land cover classified as various types of vegetation and wetlands (e.g., deciduous forest, evergreen forest, mixed forest, shrub/scrub, herbaceous, and hay/pasture), with the index ranging from 0 to 100, where higher values indicate greater coverage of green space.

#### b) ReGNN training parameters

A multi-layer perceptron (MLP) of size (20, 50, 24, 1) is embedded within a regression model. Similar to the simulated study, the network trains for 100 epochs with a batch size of 50. A dropout rate of 10% is applied during training. We use distinct learning rates for the regression coefficients (lr = 0.015) and for the MLP parameters (lr = 0.0003). To prevent overfitting, we use weight decay of 0.01 on the MLP’s parameters. 80% serve as the training set and 2% as the test set. Model optimization uses a weighted mean squared error loss function, with weights corresponding to survey weights provided by HRS:

$$\text{Weighted}\;\text{MSE}=\frac{\sum_{i}w_{i}{\left(o_{i}-{\overset{ˆ}{o}}_{i}\right)}^{2}}{\sum_{i}w_{i}}$$

where ${\text{w}}_{\text{i}}$ is the survey weight of the i^th^ sample, ${\text{o}}_{\text{i}}$ is its measured outcome variable, and ${\hat{\text{o}}}_{\text{i}}$ is its predicted outcome.

#### c) Post-training analyses

Post-training analyses follow similar procedures as in the simulated study. For SHAP values, we train ReGNN once and draw 500 background samples from the training set and 100 evaluation samples from the test set to compute SHAP values. This procedure is repeated 30 times. For each repetition, we calculate mean absolute SHAP values across the 100 evaluation samples, yielding 30 estimates per feature. These estimates are then used to compute the average and error bars of the feature importances shown in Figure 8a. For GMM, we vary the number of components from 1 to 10 and select the model with the lowest Bayesian Information Criterion (BIC).

## Figures and Tables

**Figure 1.**
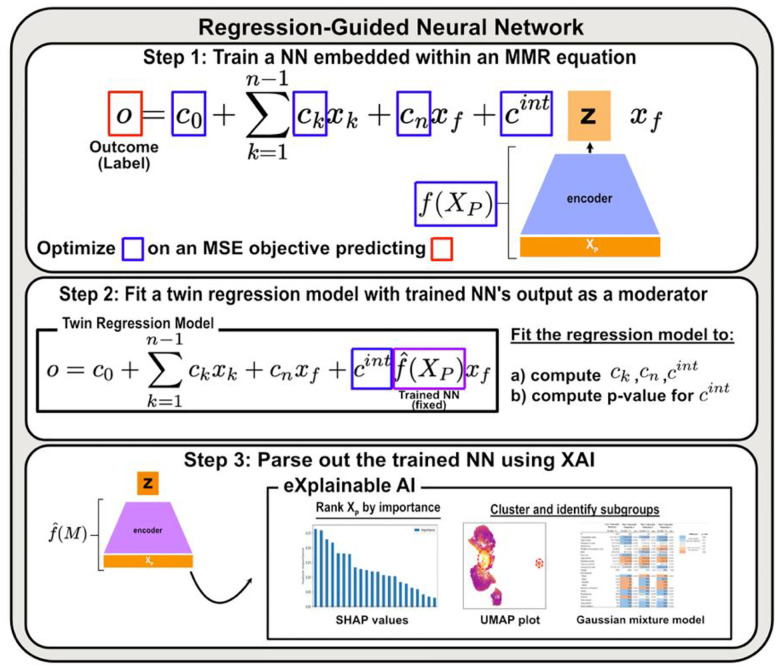
Overview of the Regression-Guided Neural Network (ReGNN) Framework. **Step 1 (top):** We set up a neural network and a regression model, where the output of the neural network is embedded as the moderator z in the regression model. The neural network takes a set of variables ${\text{X}}_{\text{P}}$ as input, representing potential sources of heterogeneity. The entire structure is trained to predict the outcome o. **Step 2 (middle):** We fit a twin regression model, or a regression model that uses the same equation as in previous step, but with the trained neural network’s output z as the moderator. The regression model computes regression coefficients, confidence intervals, and statistical significance of the interaction term. **Step 3 (bottom):** If some effect heterogeneity is detected and is found to be statistically significant from the previous step, we interpret what the neural network has learned using tools such as SHAP values and Gaussian Mixture Models (GMM) to identify which input variables contribute to the observed heterogeneity.

**Figure 1.**
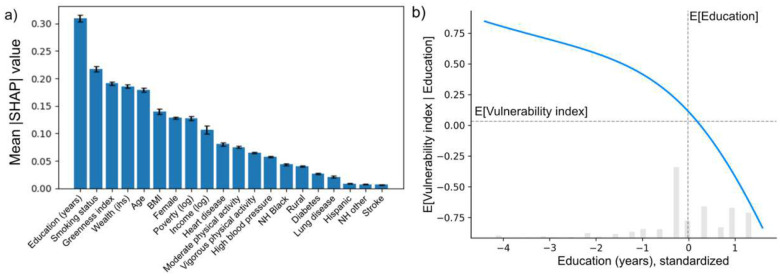
(a) Bar plot of feature importance, in descending order, of inputs to the trained ReGNN model. (b) SHAP-based partial dependence plot showing the relationship between the ReGNN-derived vulnerability index and years of education.

**Table 1.** Summary of how changing signal-to-noise ratio (SNR) affects achieved final correlation values (ρ), root-MSE and p-value of the interaction term achieved by the regression model fit using train and sensitivity sets (train to sensitivity set ratio = 80:2), and recall of ReGNN-identified vulnerable population (threshold = 0.84 quantile of z). We observe that while the p-values for model fit using the train set remain near zero for all SNR-levels, p-value achieved on the sensitivity set increases as the final correlation value decreases.

SNR	$\sigma_{\text{noise}}^{2}$	ρ	RMSE (train)	RMSE (sensitivity)	P-value (train)	P-value (sensitivity)	Recall
*5*	0.034	0.911	0.166	0.263	0.0	3.29e-27	0.69
*3*	0.057	0.877	0.196	0.341	0.0	1.68e-17	0.65
*1*	0.170	0.783	0.338	0.488	0.0	4.29e-9	0.57
*0.5*	0.341	0.663	0.444	0.765	0.0	7.64e-3	0.49
*0.3*	0.567	0.613	0.612	0.735	0.0	2.35e-3	0.45
*0.1*	1.703	0.384	0.983	1.404	0.0	0.981	0.33

**Table 2.** Top 8 features of clusters identified by GMM fit to the vulnerable subgroup (>0.9 quantile). For continuous variables, means are reported with standard deviations in parentheses. Wealth and poverty levels are reported in post-processed units using the inverse hyperbolic sine (IHS) and logarithmic (log) transformations.

	# sample	Education (years)	Smoking status	Greenness index	Wealth [ihs]	Age	BMI	Female	Poverty [log]	Vulnerability index
***#1***	233	8.62 (4.41)	Never: 0.12 Past: 0.49 Current: 0.39	27 (34)	−3.03 (6.26)	60 (9.33)	27 (6.15)	0.67	3.17 (0.55)	1.55 (0.34)
***# 2***	802	11 (2.55)	Never: 0.10 Past: 0.32 Current: 0.58	17 (25)	−3.72 (5.89)	60 (9.10)	26 (5.29)	0.66	3.24 (0.53)	1.67 (0.43)
***# 3***	584	11 (2.99)	Never: 0.0 Past: 0.0 Current: 1.0	45 (38)	−0.55 (8.21)	59 (9.39)	25 (4.59)	0.76	2.91 (0.61)	1.73 (0.47)
***# 4***	259	11 (2.85)	Never: 0.2 Past: 0.40 Current: 0.40	77 (22)	−3.14 (6.45)	62 (11)	28 (5.78)	0.65	3.03 (0.58)	1.60 (0.39)
***Total Pop*.**	18778	13 (3.13)	Never: 0.45 Past: 0.40 Current: 0.15	35 (35)	4.35 (8.57)	65 (11)	29 (6.35)	0.59	2.56 (0.76)	0.016 (0.89)

## Data Availability

The simulated dataset and accompanying code used in this study are available from the authors upon request. Data used to examine the relationship between cognition and air pollution are drawn from the Health and Retirement Study (HRS), which is restricted but can be accessed through the HRS data request process: https://hrs.isr.umich.edu. Raw environmental and contextual data, including air pollution, sociodemographic, and land cover variables, are publicly available through the U.S. Environmental Protection Agency, the American Community Survey, and the U.S. Geological Survey’s National Land Cover Database. Processed versions of these data can be accessed through https://gero.usc.edu/cbph/cdr/. Code for linking these external datasets to HRS is available from the authors upon request.

## Code availability

Code and detailed instructions for running the analyses are available on GitHub: https://github.com/njw0709/ReGNN.
